# Southern Dental Association

**Published:** 1887-09

**Authors:** 


### Editorial, Etc.

---

THE SOUTHERN DENTAL ASSOCIATION.—This Association met last week at Fortress Monroe, Old Point Comfort, and was one of the most largely attended and interesting meetings ever held. Between three and four hundred dentists were in attendance, and everything passed off in the most satisfactory manner. The late President, Dr. W. W. H. Thackston, that "Old Virginia Gentleman," can congratulate himself after presiding over such a body of dentists as congregated in 1887 at "Old Point."

We are pleased to announce the election of our old (not in years of age, but in length of friendship) friend, Dr. B. H. Catching, of Atlanta, Ga., editor of *Southern Dental Journal*, to the presidential chair for the ensuing year. Especially is this gratifying on account of the many efforts of Dr. Catching in behalf of the Southern Dental Association, and also from the fact that a vain effort was made to elect to that position a gentleman who obtained his degree of D. D. S. from the so-called Delaware (Wisconsin) College.

